# Silicon Addition Alleviates Light Stress on Seedlings: Evidence from Plantation of *Liquidambar formosana*

**DOI:** 10.3390/plants14213346

**Published:** 2025-10-31

**Authors:** Siying Cai, Minqian Zheng, Tingting Li, Youlu Hong, Yifei Chen, Zhihui Li, Junyi Lin, Xiaoli Liao, Shaofei Jin, Dexiang Zheng

**Affiliations:** 1 College of Forestry, Fujian Agriculture and Forestry University, Fuzhou 350002, China; csy0736@fafu.edu.cn (S.C.); 52404022056@fafu.edu.cn (M.Z.); 12404030012@fafu.edu.cn (T.L.); 15559113743@163.com (Y.H.); cyf123@fafu.edu.cn (Y.C.); lzhihui9801@163.com (Z.L.); 2 College of Economics and Management, Yango University, Fuzhou 350015, China; lin_juny@163.com; 3 Department of Geography, Minjiang University, Fuzhou 350108, China; liaoxl2005@126.com; 4 Observation and Research Station of Land Consolidation in Hilly Region of Southeast China, MNR, Minjiang University, Fuzhou 350108, China

**Keywords:** *Liquidambar formosana*, high-light stress, silicon fertilizer, plant resistance

## Abstract

Excessive light intensity, often resulting from anthropogenic disturbances, poses a threat to light-sensitive *Liquidambar formosana* seedlings. This study examined the effects of five light intensity levels and three silicon (Si) application rates on photosynthetic performance, oxidative stress responses, and seedling growth. Results indicated that full sunlight significantly reduced ground diameter, chlorophyll content, specific leaf area, and stomatal conductance. Meanwhile, it increased the activities of superoxide dismutase and peroxidase, and led to higher accumulation of malondialdehyde (MDA). Application of Si enhanced seedling height, biomass accumulation, and antioxidant enzyme activity under high-light conditions, while reducing MDA content, stomatal CO_2_ conductance, and transpiration rate, and maintaining a stable net photosynthetic rate. However, excessive Si (3000 mg·kg^−1^) led to decreased catalase activity, chlorophyll content, and leaf area under intense light. These findings suggest that *L. formosana* seedlings perform best under moderate shade (11,000–46,000 lx) and moderate Si application (1000–2000 mg·kg^−1^), which together mitigate photoinhibition damage. Optimal physiological responses thus require balanced Si concentrations. Further investigation is warranted to elucidate the mechanisms underlying the interactive effects of shading and Si application for improved seedling resilience.

## 1. Introduction

Light serves as a critical environmental factor for plant survival, with plant growth and physiological processes being inherently dependent on suitable light conditions [1]. Seedling regeneration, essential for the long-term sustainability of tree populations [2], may be adversely affected by climate change, undermining the effectiveness of traditional afforestation measures. A major constraint lies in the high sensitivity of young leaf cells to excessive light. Although light is essential for photosynthesis, excessive radiation induces multiple stress-response mechanisms [3].

Under prolonged high-light stress, plants initially upregulate photosynthetic capacity by enhancing the electron transport efficiency of photosystem II (PSII) or increasing Rubisco activity [4,5]. However, such responses are short-lived and may ultimately exacerbate light-induced suppression. Consequently, chloroplasts are repositioned within leaf tissues to minimize light interception [6]. Simultaneously, non-photochemical quenching (NPQ), via the xanthophyll cycle, dissipates excess light energy as heat, thereby preventing peroxidation of the PSII reaction center [6,7]. The burst of reactive oxygen species (ROS) caused by light stress further activates redox-regulatory mechanisms [8,9], including the enzymatic actions of superoxide dismutase (SOD), catalase (CAT), peroxidase (POD), and the synthesis of low-molecular-weight antioxidants.

With sustained exposure to intense light, the associated metabolic costs become apparent: ROS accumulation leads to increased mitochondrial respiration, while stomatal closure limits CO_2_ intake, disrupting the photosynthesis-respiration balance [10]. Additionally, the biosynthesis of antioxidants competes for energy and resources with pathways responsible for protein and cellulose production. This ultimately impairs overall growth, establishing a positive feedback loop that exacerbates light inhibition. This process is particularly detrimental to shade-adapted species [10], whose inherent low-light preferences increase the cost of light defense, resulting in dwarfism, reduced biomass, and regeneration failure.

In contrast, moderate shading has been shown to enhance organic matter accumulation and root development in shade-tolerant plants. For instance, seedling mass of red cone species in South Asia peaked under 60% shading [11]. Experimental shading also led to increased leaf area and chlorophyll content, reduced leaf thickness, and improved PSII efficiency and gas exchange [12]. Leaf water conductance, positively correlated with light intensity, doubled under higher light levels. However, shaded conditions typically reduce radiation, cooling needs, and transpiration demand, thus enhancing water-use efficiency [13].

The dual role of light is evident: while excessive light impairs physiological function, overly shaded environments may enhance chlorophyll content but suppress net photosynthetic rates and biomass accumulation [14]. An intermediate light level can optimize physiological performance and promote growth and reproduction [15]. However, due to the spatial and temporal heterogeneity of natural light and the economic limitations of artificial light regulation, ideal light conditions are difficult to achieve in practice, necessitating alternative strategies to mitigate strong light stress.

Silicon (Si), the second most abundant element in Earth’s crust [16], offers such a strategy. Unlike many nutrients, Si provides multiple plant benefits without toxicity, even at high concentrations [17]. Its uptake primarily occurs as monosilicic acid (Si(OH)_4_), which polymerizes into amorphous silica (SiO_2_·nH_2_O) within plant tissues, accumulating in cell walls and intercellular spaces [18,19]. Passive transport and compartmentalization prevent toxic accumulation [20]. Silicon plays a pivotal role in improving crop yield and stress resilience [21,22], often accumulating in higher quantities than potassium or calcium in species like rice and sugarcane [23].

Silicon alleviates both abiotic and biotic stressors through physical and biochemical pathways [24]. Physically, silica deposition in epidermal layers reduces water loss and impedes pathogen invasion, as observed in Cucumis melo against Acidovorax citrulli [25]. In Quercus robur, high-light exposure increased epidermal silicon, stomatal density, and trichome formation [26]. Biochemically, silicon strengthens antioxidant defense by activating enzymes such as SOD and POD. In cadmium-stressed tomato seedlings, silicon reduced levels of H_2_O_2_, superoxide, and malondialdehyde (MDA) by 40%, 34%, and 33%, respectively, alleviating oxidative damage [27]. Furthermore, in salinity-stressed mung bean, Si restored photosynthetic function by regulating Rubisco expression [28]. These mechanisms are particularly relevant for perennial trees, where prolonged stress leads to chronic metabolic decline.

*Liquidambar formosana*, an ecologically important yet low-silicon-accumulating species, has received little attention regarding silicon–light interactions, despite its widespread use in subtropical afforestation. This species is widely distributed in Southeast Asia and is a key afforestation species in southern China due to its rapid growth, fire resistance, and ecological benefits [29]. Naturally shade-tolerant, its seedlings exhibit pronounced sensitivity to high light, directly affecting survival rates. Moreover, its foliage is rich in anthocyanins, which exhibit antioxidative properties and protect against pests, ultraviolet radiation, and photooxidative stress. In other species, anthocyanin accumulation has been enhanced through silicon and light manipulation, contributing to increased resistance [30,31].

Research on the interaction between light conditions and silicon (Si) fertilization has revealed that silicon plays a multifaceted role in enhancing plant resilience under varying light and nutrient regimes. For instance, silicon application improved stomatal conductance and water relations in sorghum under water stress by enhancing hydraulic conductivity, though it showed no direct physical effect on stomatal movement under light changes [32]. Similarly, silicon dioxide nanoparticles (SiO_2_NPs) were found to mitigate low-light and nitrogen-deficiency stresses in fragrant rice by modulating antioxidant responses, nitrogen metabolism, and biomass accumulation [33]. In tomatoes, a low-silicon-accumulating species, silicon alleviated drought-induced photoinhibition and oxidative damage by optimizing light energy allocation and reinforcing chloroplast antioxidant systems [34,35].

While numerous studies have elucidated the role of silicon in mitigating abiotic stress in herbaceous crops and high-Si accumulators, its physiological and biochemical mechanisms in woody species, particularly those with low silicon uptake capacity, remain poorly explored. Furthermore, the interactive effects between shading, a common management practice in forestry, and silicon amendment are virtually unknown. This knowledge gap is critical given the contrasting life strategies and stress defense mechanisms between shade-tolerant trees and light-demanding crops. To address this, our study investigated whether and how exogenous silicon can enhance the resilience of a shade-tolerant, low-silicon-accumulating tree species to high-light stress, with the aim of providing insights into silicon-mediated stress resistance and offering practical strategies to improve afforestation success and promote forest sustainability.

## 2. Results

### 2.1. Photosynthetic Parameters Under Different Treatments

**Figure 1:**
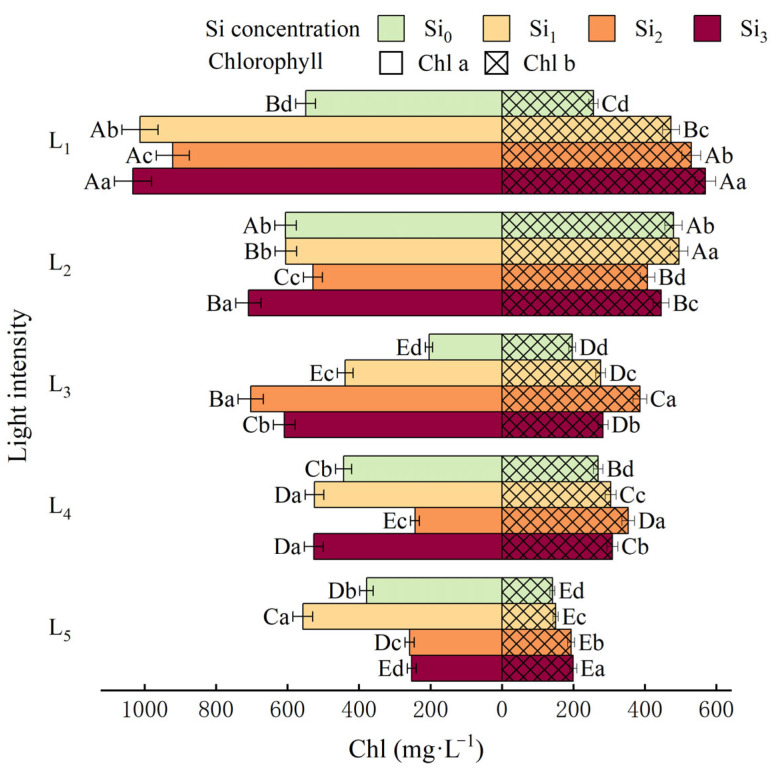
Chl a and Chl b under different treatments. Data is shown as mean ± SD (*n* = 3). Different lowercase letters indicate significant differences among shade treatments, while different uppercase letters indicate significant differences among Si treatments. The legend Si_0_–Si_3_ represents different silicon concentrations, and the labels L_1_–L_5_ indicate different light intensities. The values of these two levels are shown in Table 1. Si_0_: 0 mg/kg; Si_1_: 1000 mg/kg; Si_2_: 2000 mg/kg; Si_3_: 4000 mg/kg; L_1_: 5% of relative illumination; L_2_: 15% of relative illumination; L_3_: 40% of relative illumination; L_4_: 60% of relative illumination; L_5_: 100% of relative illumination.

**Figure 2:**
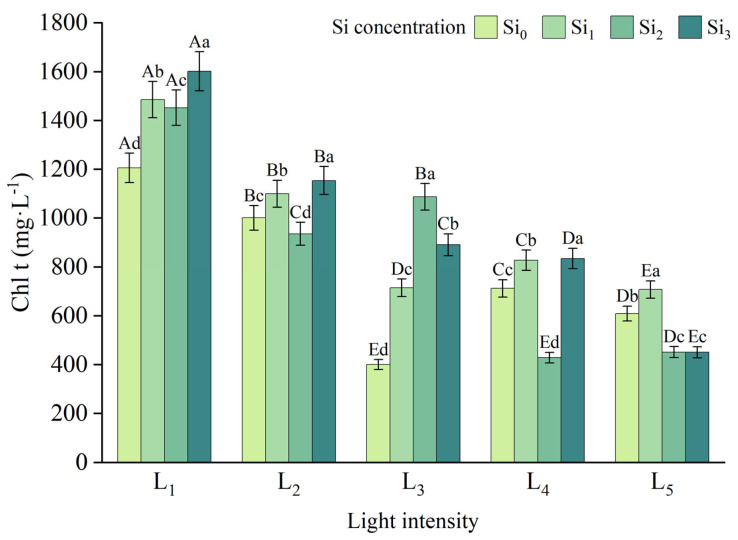
Changes in the Chl t under different treatments. Data is shown as mean ± SD (*n* = 3). Different lowercase letters indicate significant differences among shade treatments, while different uppercase letters indicate significant differences among Si treatments. The legend Si_0_–Si_3_ represents different silicon concentrations, and the labels L_1_–L_5_ indicate different light intensities. The values of these two levels are shown in Table 1. Si_0_: 0 mg/kg; Si_1_: 1000 mg/kg; Si_2_: 2000 mg/kg; Si_3_: 4000 mg/kg; L_1_: 5% of relative illumination; L_2_: 15% of relative illumination; L_3_: 40% of relative illumination; L_4_: 60% of relative illumination; L_5_: 100% of relative illumination.

Except for Si_1_, the contents of Chl a, Chl b and Chl t in the L_1_ group were significantly higher than those in the other light groups under the same Si treatment (Figure 1 and Figure 2, *p* < 0.05), and the addition of Si_3_ made the total chlorophyll content of L_1_ and L_3_ higher than that of blank group by 32.8% and 15.3%. Chl a/b reaches the maximum value at L_5_ × Si_1_ and L_5_ × Si_0_.

**Figure 3:**
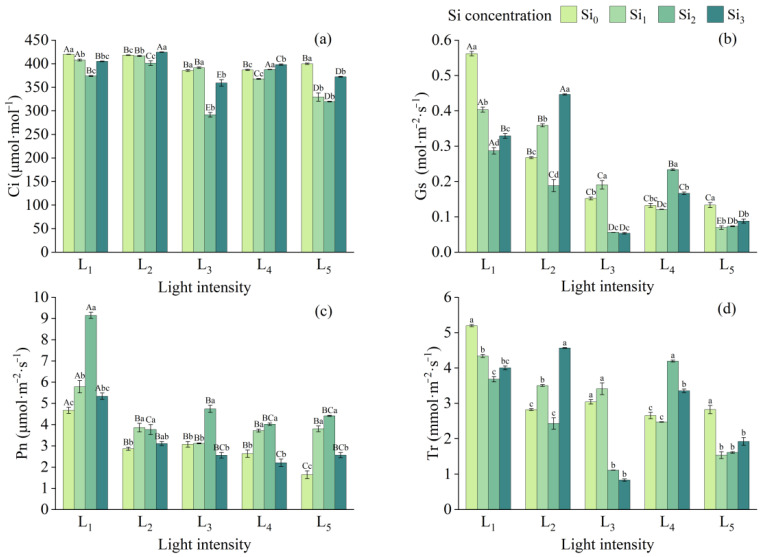
Gas exchange indicators in leaves under different treatments: (**a**) Ci, (**b**) Gs, (**c**) Pn, (**d**) Tr. Data is shown as mean ± SD (*n* = 3). Different lowercase letters indicate significant differences among shade treatments, while different uppercase letters indicate significant differences among Si treatments. The legend Si_0_–Si_3_ represents different silicon concentrations, and the labels L_1_–L_5_ indicate different light intensities. The values of these two levels are shown in Table 1. Si_0_: 0 mg/kg; Si_1_: 1000 mg/kg; Si_2_: 2000 mg/kg; Si_3_: 4000 mg/kg; L_1_: 5% of relative illumination; L_2_: 15% of relative illumination; L_3_: 40% of relative illumination; L_4_: 60% of relative illumination; L_5_: 100% of relative illumination.

Increased light intensity significantly reduced all four physiological indicators, with the most pronounced effect observed in Gs, which decreased by 76.9% from L_1_ to L_5_ under the Si_0_ treatment. In contrast, the effect on Ci was more moderate. Notably, apart from L_3_ × Si_4_ and L_4_ × Si_4_, the Pn was higher in the silicon-treated groups compared to the control, with the Si_2_ treatment showing the greatest improvement. Under the same light conditions, appropriate silicon supplementation led to changes in Ci ranging from −24.4% to +2.9%, in Gs from −64.8% to +76.4%, and in Tr from −72.6% to +61.9%, relative to the Si_0_ treatment (Figure 3).

**Figure 4:**
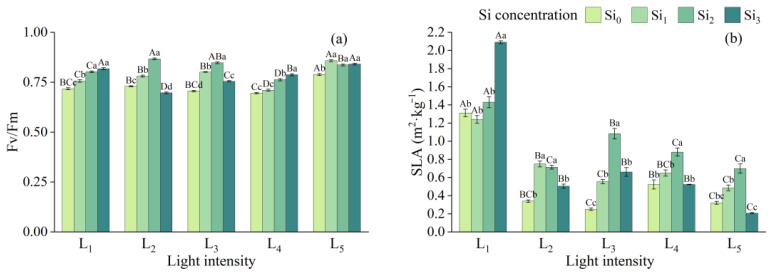
Photosynthetic functional traits under different treatments: (**a**) Fv/Fm, (**b**) SLA. Data is shown as mean ± SD (*n* = 3). Different lowercase letters indicate significant differences among shade treatments, while different uppercase letters indicate significant differences among Si treatments. The legend Si_0_–Si_3_ represents different silicon concentrations, and the labels L_1_–L_5_ indicate different light intensities. The values of these two levels are shown in Table 1. Si_0_: 0 mg/kg; Si_1_: 1000 mg/kg; Si_2_: 2000 mg/kg; Si_3_: 4000 mg/kg; L_1_: 5% of relative illumination; L_2_: 15% of relative illumination; L_3_: 40% of relative illumination; L_4_: 60% of relative illumination; L_5_: 100% of relative illumination.

Obviously, the addition of silicon promotes the Fv/Fm value in each light, which is closer to 1 at Si_2_ or Si_3_, reaching the maximum value of the same light treatment, but the value at L_2_ × Si_3_ (0.75) is far less than the overall average. With the increase in light intensity, the average value of Fv/Fm even shows an increasing trend (Figure 4).

For SLA, from L_1_ to L_5_, the overall value decreases and then increases briefly. L1 has the highest SLA of all groups, and it reaches the maximum in Si_3_ (2.09). The addition of Si_3_ under the rest of the light generally results in lower values; especially, the L_5_ group achieves the lowest SLA (0.21). When the degree of shading is low, Si_2_ usually achieves the highest value, with an additional 48.3% to 59.5% of Si_0_.

### 2.2. Antioxidant Enzyme Activities and Malondialdehyde Content Under Different Treatments

During the first two experimental stages, the SOD activity in leaves under both L_1_ and L_5_ conditions was significantly higher than under other light treatments (*p* < 0.05). Under these two light intensities, the Si_1_ and Si_2_ treatments exhibited the most pronounced stimulatory effects on SOD activity, with SOD_3_ values increasing by 1.21% and 0.74%, respectively, compared to the control. Overall, SOD activity reached its highest levels under L_4_ and L_5_. Conversely, the lowest SOD activity was observed in the Si_3_ treatment under both L_2_ and L_3_ conditions, which aligns with the pattern of lower antioxidant activity observed under moderate light conditions (L_2_ and L_3_).

**Figure 5:**
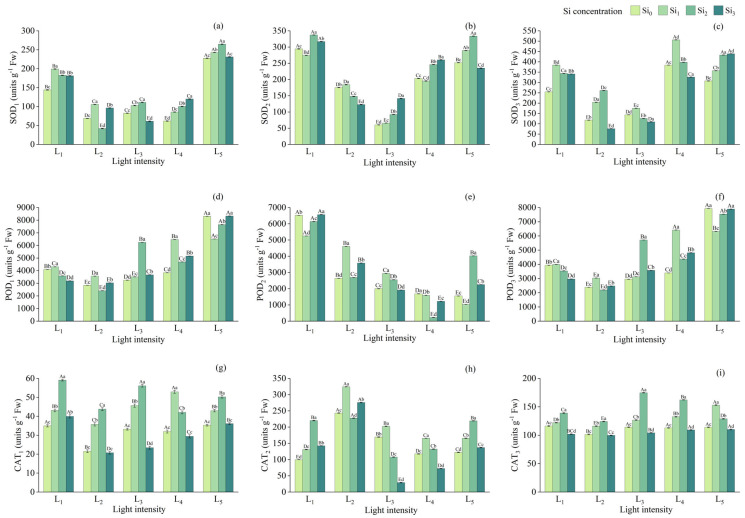
Antioxidant enzyme activities in leaves at three stages under different treatments: (**a**–**c**) SOD at 90 days, 180 days, and 270 days, respectively, (**d**–**f**) POD at 90 days, 180 days, and 270 days, respectively, (**g**–**i**) CAT at 90 days, 180 days, and 270 days, respectively. Data is shown as mean ± SD (*n* = 3). Different lowercase letters indicate significant differences among shade treatments, while different uppercase letters indicate significant differences among Si treatments. The legend Si_0_–Si_3_ represents different silicon concentrations, and the labels L_1_–L_5_ indicate different light intensities. The values of these two levels are shown in Table 1. Si_0_: 0 mg/kg; Si_1_: 1000 mg/kg; Si_2_: 2000 mg/kg; Si_3_: 4000 mg/kg; L_1_: 5% of relative illumination; L_2_: 15% of relative illumination; L_3_: 40% of relative illumination; L_4_: 60% of relative illumination; L_5_: 100% of relative illumination.

As light intensity increased from L_1_ to L_5_, POD_2_ activity showed a decreasing trend, while POD_1_ and POD_3_ exhibited the opposite pattern, with nearly identical changes between them. For CAT_1_ and CAT_2_, the Si_2_ treatment resulted in significantly higher activity under both full light (L_5_) and minimal light (L_1_) compared to other silicon levels under the same light intensity (*p* < 0.05). For the remaining light conditions, Si_1_ generally produced the highest CAT activity. In contrast, the Si_3_ treatment markedly suppressed CAT activity, with CAT_3_ remaining at a consistently low level (Figure 5).

**Figure 6:**
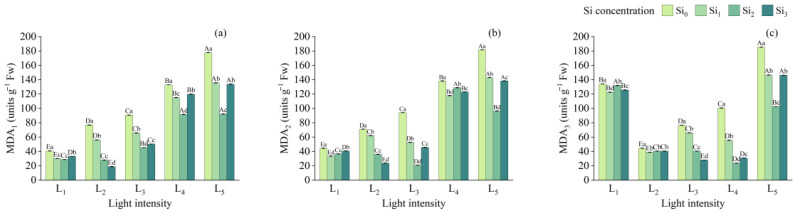
MDA content in leaves under different treatments at (**a**) 90 days, (**b**) 180 days, and (**c**) 270 days, respectively. Data is shown as mean ± SD (*n* = 3). Different lowercase letters indicate significant differences among shade treatments, while different uppercase letters indicate significant differences among Si treatments. The legend Si_0_–Si_3_ represents different silicon concentrations, and the labels L_1_–L_5_ indicate different light intensities. The values of these two levels are shown in Table 1. Si_0_: 0 mg/kg; Si_1_: 1000 mg/kg; Si_2_: 2000 mg/kg; Si_3_: 4000 mg/kg; L_1_: 5% of relative illumination; L_2_: 15% of relative illumination; L_3_: 40% of relative illumination; L_4_: 60% of relative illumination; L_5_: 100% of relative illumination.

In the first two stages, MDA content in the Si_0_ treatment increased significantly and steadily with rising light intensity and was significantly higher at both L_1_ and L_5_ compared to other treatments (*p* < 0.05). Overall, there was little difference in MDA content between silicon and non-silicon treatments under L_1_. However, under other light conditions, exogenous silicon application significantly reduced MDA levels, with Si_3_ generally resulting in the lowest values (Figure 6).

### 2.3. Growth Indicators Under Different Treatments

**Figure 7:**
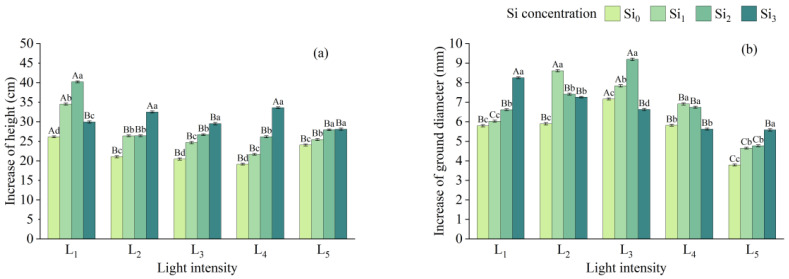
Increase in growth indicators under different treatments: (**a**) height, (**b**) ground diameter. Data is shown as mean ± SD (*n* = 3). Different lowercase letters indicate significant differences among shade treatments, while different uppercase letters indicate significant differences among Si treatments. The legend Si_0_–Si_3_ represents different silicon concentrations, and the labels L_1_–L_5_ indicate different light intensities. The values of these two levels are shown in Table 1. Si_0_: 0 mg/kg; Si_1_: 1000 mg/kg; Si_2_: 2000 mg/kg; Si_3_: 4000 mg/kg; L_1_: 5% of relative illumination; L_2_: 15% of relative illumination; L_3_: 40% of relative illumination; L_4_: 60% of relative illumination; L_5_: 100% of relative illumination.

The greatest increase in seedling height was observed under L_1_, while the maximum increase in ground diameter occurred under L_3_. In contrast, both parameters were lower under L_5_ than under other light conditions (Figure 7). Under the same shading level, the average seedling height growth in silicon-treated seedlings was 5.77% to 72.42% higher than that of the control group, with Si_2_ and Si_3_ treatments showing the most pronounced effects. However, the effect of silicon on ground diameter growth under different light intensities did not follow a clear pattern. Notably, under L_3_ and L_4_, the ground diameter growth of the Si_3_ treatment was even lower than that of Si_0_.

**Figure 8:**
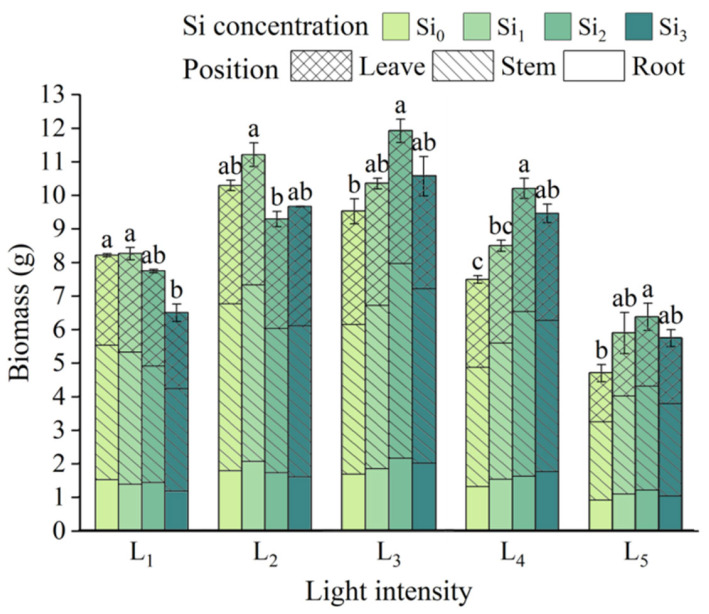
Seedling biomass allocation under different fertilization and shade treatments. Data is shown as mean ± SD (*n* = 3). Different lowercase letters indicate significant differences among shade treatments. The legend Si_0_–Si_3_ represents different silicon concentrations, and the labels L_1_–L_5_ indicate different light intensities. The values of these two levels are shown in Table 1. Si_0_: 0 mg/kg; Si_1_: 1000 mg/kg; Si_2_: 2000 mg/kg; Si_3_: 4000 mg/kg; L_1_: 5% of relative illumination; L_2_: 15% of relative illumination; L_3_: 40% of relative illumination; L_4_: 60% of relative illumination; L_5_: 100% of relative illumination.

Total seedling biomass reached a significant maximum under L_3_, while the lowest biomass was observed in the L_5_ × Si_0_ treatment. Except under L_1_ and L_2_, silicon addition consistently increased biomass across all light treatments, with the highest values in each group typically achieved under the Si_2_ treatment (Figure 8).

### 2.4. Correlations Between Photosynthetic, Antioxidant, and Biomass Parameters

**Figure 9:**
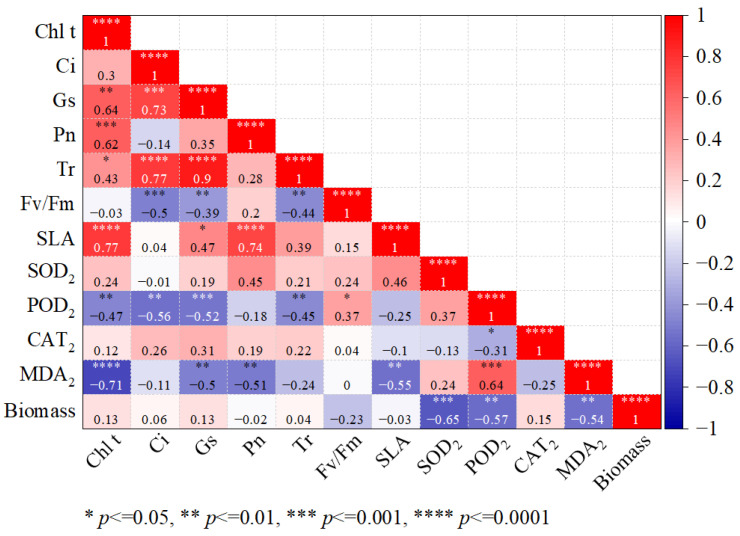
Correlation heatmap of photosynthetic, antioxidant, and biomass parameters. Cht: total chlorophyll; Ci: intercellular CO_2_ concentration; Gs: stomatal conductance; Pn: net photosynthetic rate; Tr: transpiration rate; Fv/Fm: the maximum photochemical efficiency of PSII; SLA: Single leaf area; SOD: superoxide dismutase; POD: peroxidase; CAT: catalase; MDA: malondialdehyde; Biomass: biomass of the leaf.

According to Figure 9, Chl t was significantly positively correlated with Pn (r = 0.621, *p* < 0.001). Ci was significantly positively correlated with Tr (r = 0.767, *p* < 0.001). POD_2_ was significantly negatively correlated with MDA_2_ (r = −0.639, *p* < 0.001). Meanwhile, MDA_2_ was significantly negatively correlated with total biomass (r = −0.541, *p* < 0.001).

## 3. Discussion

### 3.1. Effects of Shade and Silicon Addition on Chlorophyll Content in Liquidambar formosana Leaves

The effects of shading and Si supplementation on chlorophyll content in the leaves of *Liquidambar formosana* seedlings were investigated. Chlorophyll plays a vital role in capturing light energy and converting it into chemical energy, thereby directly influencing photosynthetic efficiency. Its content and photosynthetic characteristics are responsive to abiotic environmental factors such as light, CO_2_ concentration, and temperature. Within a physiological range, increased light intensity typically promotes chlorophyll synthesis to enhance light-harvesting efficiency for photosynthesis.

**Figure 10:**
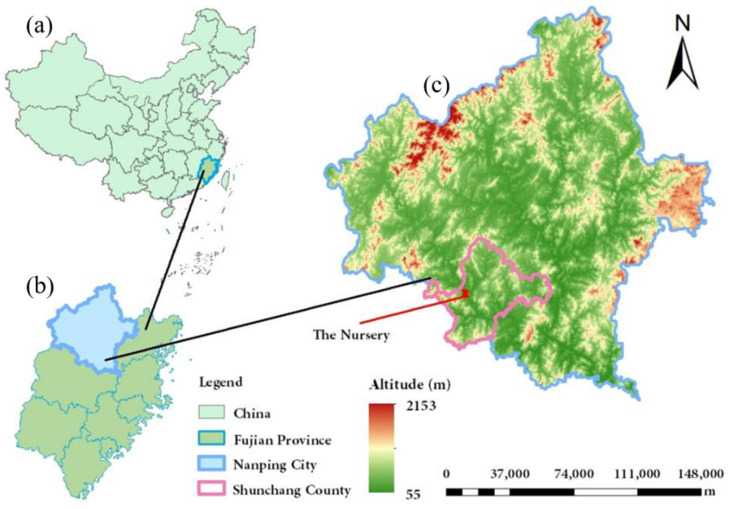
Map of the study located. (**a**) China, (**b**) Fujian Province, (**c**) Nanping City. The red polygon in (**c**) refers to the nursery site is located.

Under low light conditions (L_1_), seedlings exhibited a compensatory increase in chlorophyll content (Figure 10), likely serving to counteract light limitation by enhancing light absorption. This observation aligns with the findings of Huang in Boehmeria nivea, where shaded conditions led to significant increases in chlorophyll a + b content per unit leaf dry weight, albeit with smaller increments under short-term shading [36]. In contrast, high light intensities (L_4_–L_5_) resulted in photoinhibition, as evidenced by decreased chlorophyll content and elevated SOD and POD activity across all three measurement periods (Figure 1 and Figure 2), indicating the induction of oxidative stress. These results suggest that the absence of shading is detrimental to seedling growth.

The observed light–chlorophyll–antioxidant interaction is consistent with broader plant stress adaptation mechanisms. For instance, in Populus hybrids subjected to intense light, chlorophyll a/b ratios declined markedly, while SOD and POD activities increased to mitigate oxidative damage [37], paralleling the response observed under L_5_. Shade-adapted species such as Dendrobium huoshanense typically accumulate more chlorophyll b to optimize light capture; however, insufficient light can reduce photosynthetic efficiency and delay ROS scavenging [38]. Moderate light levels (L_2_–L_3_) appear to provide an optimal balance. In rice mutants with reduced chlorophyll b content, moderate light enhanced PSII stability by upregulating SOD and CAT activity, thereby reducing H_2_O_2_ accumulation and photodamage [39]. Similarly, Medicago sativa under moderate heat stress maintained chlorophyll content through increased POD and CAT activity, whereas excessive stress damaged thylakoid membranes and impaired carbon fixation [40].

These findings suggest that moderate light intensities promote chlorophyll synthesis while minimizing oxidative overload, as demonstrated in *L. formosana* seedlings under L_2_–L_3_. The role of light-harvesting chlorophyll a/b-binding proteins (Lhcs) may further underpin this mechanism. Under drought stress, Lhcs have been shown to activate ABA signaling, which stabilizes chlorophyll content and enhances antioxidant enzyme activity [40]. For example, Arabidopsis mutants overexpressing Lhcb1 retained higher chlorophyll levels and exhibited elevated SOD activity under high light, resembling the adaptive responses observed under L_2_–L_3_ in this study. Additionally, research on Triticum aestivum indicates that moderate light optimizes NPQ, allowing excess energy to be dissipated without compromising CO_2_ assimilation [41]. Collectively, these findings support the hypothesis that the synergistic regulation of chlorophyll retention and antioxidant activity under L_2_–L_3_ light conditions constitutes a conserved adaptive strategy that maintains photosynthetic efficiency while mitigating oxidative stress.

### 3.2. Positive Effect of Silicon Addition on Photosynthetic Efficiency of Liquidambar formosana

The beneficial role of silicon in enhancing photosynthetic performance under varying light conditions has been attributed to conserved physiological mechanisms across plant species. Notably, the lowest Pn was consistently observed in the absence of silicon (Si_0_), underscoring the importance of Si addition under light stress. Improvements in photosynthesis associated with Si may involve optimized chloroplast development [42], stomatal regulation, enhanced enzyme activity, antioxidant defense, and transcriptional regulation [38].

In this study, the decreases in Gs, Ci, and Tr occurred concurrently, whereas Pn increased, indicating that the observed photosynthetic enhancement was likely influenced more by non-stomatal factors (e.g., chlorophyll content, PSII efficiency) than by stomatal conductance alone. Meanwhile, an appropriate concentration of Si (Si_2_) significantly increased chlorophyll content and maximum quantum yield of PSII (Fv/Fm). Silicon was also reported to alter the mechanical properties of mesophyll cells through silica-hydroxyl cross-linking in cell walls, thereby indirectly improving the substrate binding efficiency of Rubisco [43,44].

Previous studies have demonstrated that Si significantly enhances Pn under low light, likely due to induced morphological adaptations. Si supplementation promoted vertical leaf orientation via cell wall silicification, increasing SLA, improving light distribution within the canopy, and enhancing light capture in lower leaves [45]. Additionally, Si-treated plants exhibited higher carotenoid content, further supporting enhanced photoreaction efficiency by enabling the absorption of a broader light spectrum, including blue-violet light. Based on light distribution models [46], Si-induced SLA increases may raise canopy transmittance by 25%, which may account for the greater Pn enhancement in lower leaves (+25%) compared to upper leaves (+8%).

Under high light conditions (L_5_), Si treatment led to a significant reduction in chlorophyll content. This may indicate resource reallocation, where Si-treated plants invested relatively more in carbon fixation and ROS detoxification than in chlorophyll synthesis, although further biochemical evidence is needed. MDA levels were consistently lower in Si-treated groups compared to controls, while antioxidant enzyme activities exhibited an initial increase followed by a variable decline. This aligns with Liang’s findings [47] that Si-induced activation of specific SOD isoenzymes (e.g., Cu/Zn-SOD) enhances superoxide clearance.

Furthermore, Si may promote carbon fixation over chloroplast biosynthesis by modulating carbon allocation, as indicated by a 15% increase in soluble sugar content. Gao [48] also demonstrated that Si helps maintain the structural integrity of the electron transport chain by stabilizing PSII protein D1, sustaining Fv/Fm values above 0.75 under strong light. However, excessive Si application (Si_3_) was associated with reduced Pn, likely due to inhibition of Gs and Ci and limited additional antioxidant benefits, suggesting a concentration threshold for optimal Si efficacy [49,50]. While moderate silicon deposition strengthens cell walls, over-silicification could reduce cell elasticity, impede gas exchange, and hinder cell expansion [18]. The active uptake and polymerization of silicon at high concentrations may divert energy and resources away from other critical processes such as photosynthesis and repair mechanisms, particularly under extreme stress [51]. These factors highlight the importance of identifying species-specific and environment-specific optimal silicon dosages to avoid detrimental effects.

### 3.3. Effect of Silicon Addition on Growth Promotion of Seedlings Under Stress

Light is a critical environmental factor influencing plant survival, growth, and reproduction, directly affecting functional traits. Consequently, morphological indicators such as seedling height, ground diameter, and biomass vary with spatial heterogeneity and serve as proxies for assessing seedling quality and stress intensity. Under L_2_ and L_3_ conditions, seedlings exhibited a lower increment in height and a greater increment in ground diameter, resulting in a compact morphology. In contrast, seedlings under L_1_ were taller and slenderer. This trend is consistent with previous reports on morphological responses of *Cunninghamia lanceolata* to varying light intensities [51].

To increase light acquisition, seedlings grown under shaded environments tend to allocate more resources to height growth, thereby enhancing their competitive ability for light capture [52]. However, biomass accumulation was greater under L_2_ and L_3_, and Si supplementation at all concentrations significantly promoted both height and diameter growth under identical light conditions.

At the cellular level, Si-induced synergism between cell wall silicification and expansion has been reported. Silicon deposition in the cell wall leads to the formation of a double-layered “Si–epidermis” structure, which enhances mechanical strength and structural stability. In maize, Si treatment increased plant height and leaf area by 12.3% and 15.8%, respectively, independent of the acid-growth mechanism associated with plasma membrane H^+^-ATPase activity, but possibly regulated via expansin activity [53]. For seedlings, lateral growth is also associated with enhanced water uptake capacity. Similarly, Si-induced cell wall silicification in rice has been shown to reduce transpiration rate and decrease leaf H_2_O_2_ and MDA content by 30–45%, thereby maintaining cellular water balance [54].

The nonlinear response of SLA to light and silicon (Figure 4b) reflects a strategic trade-off between light capture efficiency and photoprotective investment. Under deep shade (L_1_), the significantly higher SLA (*p* < 0.001) represents a classic morphological adaptation to maximize light interception per unit biomass. The subsequent decline in SLA with increasing light intensity suggests a shift in resource allocation towards leaf thickening and palisade tissue development, which enhances photoprotection but reduces light capture efficiency per unit mass. This morphological shift is corroborated by the strong positive correlation between SLA and photosynthetic pigments (Chl t: r = 0.768, *p* < 0.001), indicating that thinner leaves (high SLA) are associated with greater light-harvesting capacity.

The resurgence of SLA under moderate light (L_3_) with Si_2_ treatment is particularly intriguing. It indicates that silicon supplementation may facilitate a more efficient and resilient leaf morphology, optimizing both light capture and structural integrity without necessitating excessive investment in defensive structures [46]. This silicon-mediated optimization is likely linked to the observed reduction in oxidative stress. Our correlation analysis reveals a significant negative relationship between SLA and the lipid peroxidation marker MDA (MDA_2_: r = −0.548, *p* < 0.001), suggesting that leaves with higher SLA under Si amendment are not more vulnerable to oxidative damage. On the contrary, silicon may enhance the biochemical photoprotective capacity, as supported by the strong negative correlation between key antioxidant enzymes (e.g., POD_2_) and MDA (POD_2_ vs. MDA_2_: r = −0.639, *p* < 0.001). Therefore, silicon appears to decouple the traditional trade-off, allowing for the maintenance of a more acquisitive leaf morphology (higher SLA) while concurrently bolstering the antioxidant system to manage the associated oxidative pressure.

These findings support Hattori’s model [55] of Si-mediated enhancement of cell wall rigidity, in which Si deposition promotes morphogenesis through both physical fortification and chemical signaling. Additionally, Si mitigates the adverse effects of photoinhibition on carbon metabolism by stabilizing photoassimilates, further highlighting its pleiotropic regulatory roles. It should also be noted that the effects of Si on biomass allocation may vary depending on plant species [45].

## 4. Materials and Methods

### 4.1. Study Area

This study was conducted in Shunchang County, Nanping City, Fujian Province, southeastern China (117°29′–118°14′ E, 26°38′–27°12′ N) (Figure 10). The region has a subtropical monsoon climate, with an average annual temperature of 20 °C and an average annual precipitation of 1359 mm. The nursery site is at an altitude of 134 m, with basic soil physicochemical properties including pH 6.92, organic matter 0.14 g/kg, available phosphorus 0.03 g/kg, and readily available potassium 0.08 g/kg.

### 4.2. Experimental Design

One-year-old *Liquidambar formosana* seedlings with similar height, diameter at breast height (DBH), and canopy structure were selected for the experiment. A total of 200 seedlings were transplanted into plastic cylindrical pots (bottom diameter: 20 cm; height: 30 cm), each containing 3 kg of soil with a measured pH of 5.0 and an available phosphorus content of 5 mg·kg^−1^. Throughout the experimental period, seedlings were regularly irrigated, and no additional exogenous substances were applied.

The experimental design included five light intensity treatments: 100% (full sunlight), 60%, 40%, 15%, and 5% of ambient light. The full sunlight group was placed outdoors under natural light conditions, while the remaining treatments were conducted under shade chambers constructed using iron frames covered with different matching nylon meshes of single-layer 40 mesh, single-layer 60 mesh, 60 mesh + 40 mesh and double-layer 80 mesh to achieve the desired shading levels. Each shade chamber measured 6 m × 4 m × 3 m and was spaced 3.0 m apart to minimize mutual interference among treatments.

Silicon treatments were applied using a water-soluble sodium metasilicate (Na_2_SiO_3_·5H_2_O, analytical grade) with a SiO_2_ content ≥ 50%. Root application was employed instead of foliar spraying to enhance fertilizer uptake efficiency and avoid potential foliar damage caused by high-concentration spraying, particularly under variable weather conditions. Additionally, the physical characteristics of the leaves—being thin, leathery, and pubescent—were considered less conducive to efficient foliar absorption. Since silicon is not readily fixed in the soil, root fertilization allowed for better control of silicon uptake.

**Figure 11:**
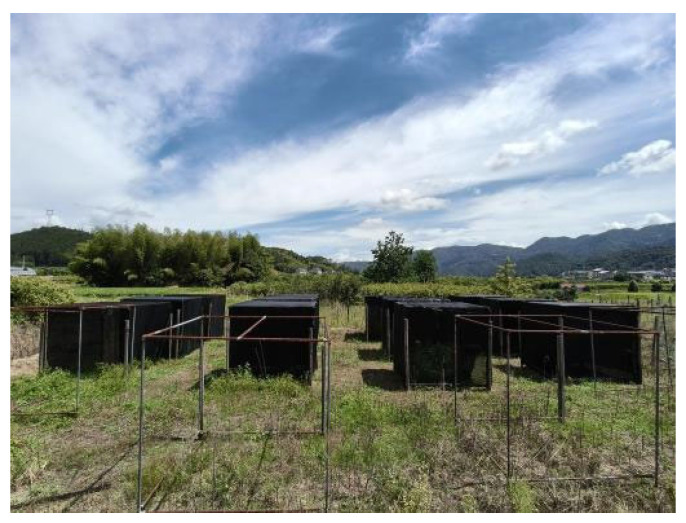
Photographs of experimental treatments. The two experimental frame on the front without shade are the group of 100% of relative illumination, while the other frames have different sunshades placed on them according to the experimental design.

A total of 3 applications were made at 15-day intervals, starting 1 week after transplanting. Each application delivered the respective silicon concentration (0, 1000, 2000, or 4000 mg·kg^−1^ soil) in 200 mL of aqueous solution per pot. The experiment consisted of two variables (light intensity and silicon concentration) in a factorial design with three replicates, comprising 20 treatment combinations with six seedlings per treatment (Table 1, Figure 11). To track the dynamic physiological responses, we measured the key indicators (antioxidant enzymes and malondialdehyde content) at 3 critical stages. These stages were 90 days, 180 days, and 270 days after the treatment, respectively.

**Table 1:** Treatment of four silicon fertilizer concentration gradients and five light intensity gradients.

Levels of Si Concentration	Si Concentration (mg/kg)	Levels of Light Intensity	Illumination (lx)	Relative Illumination (%)
Si_0_	0	L_1_	3813.28 ± 289.58	5
Si_1_	1000	L_2_	11,695.45 ± 485.21	15
Si_2_	2000	L_3_	31,006.29 ± 534.82	40
Si_3_	4000	L_4_	46,123.75 ± 915.26	60
-	-	L_5_	76,910.37 ± 1025.23	100

### 4.3. Measurement of Growth Indicators

Seedling height was measured using a tape measure, while ground diameter was determined with an electronic vernier caliper. For biomass assessment, entire seedlings were carefully uprooted, cleaned, and subsequently divided into three components: roots, stems, and leaves. These components were initially subjected to a temperature of 105 °C for 30 min to eliminate microbial activity. Thereafter, they were dried at 65 °C until a constant weight was achieved. The dry weight of each component was recorded, and the corresponding biomass was calculated accordingly.

### 4.4. Testing of Antioxidant Enzym Activities and Malondialdehyde Content

0.2 g of fresh leaves were placed in phosphate-buffered solution and thoroughly ground under an ice bath. Then, they were transferred to a 5-milliliter centrifuge tube and centrifuged at 4 °C and 10,000× *g* for 20 min. The supernatant was used as the enzyme solution. The activity of SOD was determined using the nitroblue tetrazolium (NBT) photoreduction method. Peroxidase POD activity was measured based on the guaiacol oxidation method, while catalase CAT activity was assessed via ultraviolet (UV) absorption. The malondialdehyde MDA content was quantified using the thiobarbituric acid (TBA) method [35]. The Spectramax ID5 microplate reader was used to measure the absorbance of the reaction system.

### 4.5. Testing of Photosynthetic Efficiency

The absorbance of the chlorophyll extract was measured at 663 nm and 645 nm using a microplate reader after fresh chlorophyll was extracted with 95% ethanol. Based on the absorbance values, the contents of chlorophyll a (Chl a), chlorophyll b (Chl b), and total chlorophyll (Chl t) were calculated accordingly.

For the determination of the maximum photochemical efficiency of PSII (Fv/Fm), the same leaf area used for chlorophyll pigment measurement was selected, and measurements were conducted between 9:00 and 11:00 a.m. The leaves were dark-adapted for 20 min prior to fluorescence measurements. Maximal fluorescence (Fm) and variable fluorescence (Fv) were then recorded using a FluorCam chlorophyll fluorescence imaging system in broadleaf seedlings.

Single leaf area (SLA) and total leaf area were calculated based on leaf length and width measurements obtained using ImageJ software (Version 1.54p). For each treatment, 3 leaves from equivalent positions were selected for analysis.

On clear and sunny days, gas exchange parameters including net photosynthetic rate (Pn), stomatal conductance (Gs), transpiration rate (Tr), and intercellular CO_2_ concentration (Ci) were measured on fully expanded, healthy leaves selected from the middle to upper canopy of representative plants in each treatment group using a LI-6400XT portable photosynthesis system.

### 4.6. Statistical Analysis

A two-way analysis of variance (ANOVA) was performed to test interactions between treatment levels and assess data significance, validity, and reliability. One-way ANOVA and interaction analysis were applied to test the significance of differences in the traits. Tukey’s honest significant difference (HSD) post hoc test was conducted for multiple comparisons when factors or interactions showed significance at a level of *a* = 0.05. These analyses were performed using the R programming software (version 4.3.2).

Additionally, Pearson’s correlation analysis was conducted to examine the linear relationships between measured photosynthetic, antioxidant, and biomass parameters. The correlation coefficients and their significance levels (*p*-values) were calculated, and a correlation matrix heatmap was generated for visualization using the R programming software (version 4.3.2).

## 5. Conclusions

The mitigating effects of exogenous silicon application on the growth and physiological responses of *Liquidambar formosana* seedlings under high light stress were investigated. It was observed that silicon induced adaptive responses to high light and enhanced stress resistance; however, higher concentrations of silicon were found to be less effective. The alleviation of strong light stress by silicon was attributed to its roles in physical protection, biochemical modulation, and physiological regulation. Nonetheless, the defense mechanisms inherent to the seedlings, in conjunction with silicon supplementation, were insufficient to fully counteract the detrimental effects of intense light. The observed variations under different light intensities highlighted the shade tolerance of *Liquidambar formosana* seedlings. Our findings demonstrate that the mitigating effect of silicon on *Liquidambar formosana* seedlings is highly dependent on light intensity, with an optimal combination of moderate shading (40–60% light) and moderate silicon application (1000–2000 mg·kg^−1^) yielding the best physiological and growth responses.

## Data Availability

The data presented in this study are available on request from the corresponding author.
